# Association between Uric Acid and Metabolic Syndrome in Qazvin Metabolic Diseases Study (QMDS), Iran: Association between Uric Acid and Metabolic Syndrome

**DOI:** 10.5539/gjhs.v5n1p155

**Published:** 2012-11-15

**Authors:** Amir Ziaee, Neda Esmailzadehha, Azam Ghorbani, Saeed Asefzadeh

**Affiliations:** 1 Metabolic Diseases Research Center, Qazvin University of Medical Science, Qazvin, Iran; 2 Research Center for Social Determinants of Health, Qazvin University of Medical Sciences, Qazvin, Iran

**Keywords:** prevalence, hyperuricemia, logistic regression, association, uric acid

## Abstract

**Background:**

The prevalence of Metabolic Syndrome (MS) has been increasing worldwide. Although Uric Acid (UA) Levels are often increased in subjects with MS, it is still unclear whether uric acid plays a causal role for MS or is a marker. The purpose of this study was to examine the association between UA and the MS in Qazvin, Iran.

**Methods:**

529 men and 578 women aged 20 – 78 years attended in cross sectional study from September 2010 to April 2011 in Qazvin, Iran. The criteria proposed by new joint Interim societies (JIS) were applied for diagnosis of MS. Hyperuricemia was defined as UA ≥ 7 mg/dL in men and UA ≥ 6 mg/dL in women. Logistic regression analysis was performed to evaluate the relationship between UA quartiles and MS.

**Results:**

The prevalence of MS was found to be 39.3%. Prevalence of hyperuricemia was 8.4% in males and 4.1% in females (P=0.004). Mean UA level was higher in males than in females (P<0.001). UA levels increased significantly with an increasing number of MS components in both genders. Prevalence of MS increased across UA quartiles in females; however the increasing trend began from second quartile in males. Using the lowest quartile of UA level as a reference, there were no significant association between UA quartile groups and MS.

**Conclusion:**

This study showed that UA levels are not an appropriate predictor of MS in Iranian population. More longitudinal studies are necessary to confirm the role of UA in MS occurrence.

## 1. Introduction

The metabolic syndrome (MS) refers to a cluster of risk factors, including abdominal obesity, high blood pressure, dyslipidemia and increased plasma glucose (Eckel, Grundy, & Zimmet, 2005). MS is a modern epidemic that is strongly associated with the development of cardiovascular disease and diabetes mellitus (Wilson et al., 2005; Tsouli et al., 2006). The prevalence of MS has been increasing worldwide (Meshkani, Zargari, & Larijani, 2010). In an Iranian study, the prevalence of MS was 30.1% (Azizi et al., 2003). Increasing evidence suggests that uric acid (UA) level may play a role in the MS (Chiou et al., 2010; Nan et al., 2008; Sui et al., 2008).

Large epidemiological studies have established the association of increased serum uric acid levels with MS and its individual components (Lin, Tsai, & Hsu, 2006; Onat et al., 2006; Choi & Ford, 2007; Reimann et al., 2008; Liu, Chang, & Chen, 2010; Saggiani et al., 1996; Bonora et al., 1996; Athyros et al., 2005; Zimmet et al., 1994). Additionally, UA level has been associated with increasing numbers of MS components (Tsouli et al., 2006; Lin, Tsai, & Hsu, 2006; Schmidt et al., 1996). Although UA Levels are often increased in subjects with MS (Yoo et al., 2005; Schmidt et al., 1996), none of the proposed definitions include UA levels in its criteria (Tsouli et al., 2006). It is still unclear whether uric acid plays a causal role for MS or is a marker (Lu et al., 2012).

The prevalence of hyperuricemia has been increased in recent years, in developing countries (Conen et al., 2004). Previous studies demonstrated that increased UA are markers of increased cardiovascular risk independently (Gagliardi, Miname, & Santos, 2009; Montalcini et al., 2007). Given the high prevalence of MS in Iranians, the purpose of this was to examine the association between UA and the MS in Qazvin, Iran.

## 2. Methods

### 2.1 Subjects

This study was a cross sectional population based study that was performed on a representative sample of residents of mindoodar district of Qazvin which is located 150 km northwest of Tehran, the capital city of Iran. The ethics committee of Qazvin University of medical sciences approved the study.

All households of the district had health profiles at minoodar health center and the sampling unit was the household. The district was divided into four main clusters with respect to the population size. Inclusion criteria were age > 20 years, owning an apartment in the area and residence for at least next 5 years. People aged > 20 years in every household were selected by multistage cluster random sampling methods. Subjects were invited by phone call to attend the study at the health center and after face to face explanation of the study details, they were free to participate. All subjects gave their written informed consent. 1107 subjects eligible for the study were selected and they were evaluated from September 2010 to April 2011.

### 2.2 Data Collection

Social and demographic data were self-reported in the questionnaire given to the subjects. An organized questionnaire including past medical history, family medical conditions, current medication and physical examination was initially prepared from a literature review. Face and content validity of the questionnaire were reviewed and approved by five experts. Two general practitioners filled out the questionnaires. Anthropometric data were obtained after a 12 – 14 hours over night fast. Body weight was measured while subjects were lightly clothed to the nearest 0.1 kg using a Seca scale, Germany. Height was measured in a barefoot standing position to an accuracy of 0.5 cm using tape meter while the subjects look straight forward. Body mass index (BMI) was calculated as weight (kg) divided by the height (m) squared. Waist and hip circumference were measured to the nearest 0.1 cm using a flexible, non-elastic measuring tape without any pressure on the tissue. The waist circumference (WC) was measured halfway between the costal margin and the iliac crest at the end of normal expiration. The hip circumference (HC) was considered as the maximal circumference over the great femoral trochanters. Waist–to–hip ratio (WHR) was calculated as WC divided by HC. Blood pressure (BP) was measured three times – on a single occasion – in a seated position using a mercury sphygmomanometer after a 15 min rest. There was an at least 2 minute interval between last two measurements and their average value was registered as subject’s BP. If an abnormal value was obtained, then another measurement was taken after a 30 minute rest.

### 2.3 Laboratory Tests

A venous blood sample of subject was taken after a 12 – 14 hour overnight fast. All the samples were analyzed at the same laboratory on the day of blood collection. Blood levels of glucose, insulin, total cholesterol (Chol), high density lipoprotein cholesterol (HDL-C), low density lipoprotein cholesterol (LDL-C), triglycerides (TGs), creatinin (Cr) and uric acid (UA) were measured in all subjects.

A Hitachi 704 auto-analyzer with GOD-PAP method and reagent (Parsazmun Company, Tehran, Iran) was used to measure fasting blood glucose (FBS); Mean intra- and interassay coefficients of variation (CVs) were 1.28 and 0.84, respectively. An oral glucose tolerance test (OGTT) was performed on every subjects who had never been diagnosed with diabetes. Impaired glucose tolerance was defined as a glucose level greater than 140 mg/dL (7.8 mmol/L) but less than 200 mg/dL (11 mmol/L) at two hours. Insulin levels were measured by ELISA using reagent (Monobind Company, USA). A within-run precision CV was 4.9 and total precision CV was 4.9. A Hitachi 704 auto-analyzer with CHOD-PAP and reagent (Parsazmun Company) was used to measure cholesterol; Mean intra- and interassay CVs were 0.61 and 1.22, respectively. A Hitachi 704 auto-analyzer with Immunoturbidimetric and reagent (Parsazmun Company) was used to measure HDL-C and LDL-C; Mean intra- and interassay CVs for HDL-C were 0.73 and 1.8, respectively; for LDL-C they were 0.63 and 1.29, respectively. A Hitachi 704 auto-analyzer with GPO-PAP and reagent (Parsazmun Company) was used to measure TGs; Mean intra- and interassay CVs were 1.82 and 1.04, respectively. A Hitachi 704 auto-analyzer with Uricase and reagent (Parsazmun Company) was used to measure UA; Mean intra- and interassay CVs were 1.18 and 1.13, respectively.

### 2.4 Definitions

Insulin resistance (IR) was estimated by the homeostatic model assessment (HOMA-IR), as fasting serum insulin (μIU/ml) × fasting plasma glucose (mmol/L) /22.5 (Matthews et al., 1985). Using the last Joint Interim Society criteria, (Alberti et al., 2009) MS was identified when at least three of the following conditions were met: WC ≥ 94 cm in men or ≥ 80 cm in women, serum triglycerides ≥ 150 mg/dl or receiving treatment for hypertriglyceridemia, HDL < 40 mg/dl for men and < 50 mg/dl for women and/or receiving treatment for reduced HDL (e.g. Atorvastatin), Fasting plasma glucose ≥ 100 mg/dl (includes diabetes) and systolic blood pressure (SBP) ≥ 130 mmHg or diastolic blood pressure (DBP) ≥ 85 mmHg and/or receiving treatment for hypertension. Hyperuricemia was defined as UA ≥ 7 mg/dL in men and UA ≥ 6 mg/dL in women (Lu et al., 2012; Gonçalves et al., 2012).

### 2.5 Data analysis

Kolmogorov Smirnov test was used to examine the normality of variables of interest. Data were recorded as mean plus or minus standard deviation (SD) for normally distributed variables or as median values (minimum – maximum) for non–normally distributed variables. Categorical variables were analyzed by chi square test, T-test was used for analysis of continuous variables and non-normally distributed variables were compared by Mann Whitney U test. The relationship between UA level and other variables including MS components were assessed by Pearson’s correlation coefficients. Logistic regression analysis was performed to evaluate the relationship between UA quartiles and MS. Various variables were considered as the potential confounding factors in five models. P-values less than 0.05 were considered statistically significant.

## 3. Results

The study was performed for a total of 529 men and 578 women aged 20- 78 years (40.08 ± 10.33). Table 1 presents clinical and biochemical characteristics of the subjects. BMI, WC and WHR were significantly higher in females than in males. Prevalence of the MS was 39.3% of according to the JIS criteria. Overall prevalence of hyperuricemia was 6.2%. Prevalence of hyperuricemia was 8.4% in males and 4.1% in females. (P= 0.004) Mean UA level was higher in males than in females. (P<0.001)

**Table 1:** Clinical and biochemical characteristics of the study subjects

	Total	Men	Women	p-value
**Age** (year)^a^	40.08 ± 10.33	42.31 ± 10.56	38.02 ± 9.69	*< 0.001*
**BMI** (kg/m^2^)^a^	25.97 ± 4.50	25.15 ± 3.71	26.73 ± 5	*< 0.001*
**WC** (cm)^a^	89.52 ± 10.57	92 ± 9.25	87.26 ± 11.18	*< 0.001*
**WHR**^a^	0.846 ± 0.076	0.886 ± 0.060	0.810 ± 0.071	*< 0.001*
**SBP** (mmHg)^a^	112.35 ± 17.76	115.44 ± 17.09	109.54 ± 17.90	*< 0.001*
**DBP** (mmHg)^a^	71.59 ± 11.85	73.75 ± 11.46	69.62 ± 11.87	*< 0.001*
**Fasting blood glucose** (mg/dL)^b^	93.5 (73 - 444)	95 (75.3 - 444)	92 (73 - 353.3)	*< 0.001*
**2 hrs Blood glucose** (mg/dL)^b^	104.1 (60 – 551.5)	99 (63 – 491)	107 (60 – 551.5)	*< 0.001*
**Fasting Insulin** (μIU/mL)^b^	11.1 (0.6 - 135)	10.9 (1.5 - 135)	11.3 (0.6 - 87.9)	*NS*
**TGs** (mg/dL)^b^	121 (40 - 873)	134.5 (41 - 873)	108.5 (40 - 467)	*< 0.001*
**Total cholesterol** (mg/dL)^a^	183.29 ± 39.34	184.38 ± 39.20	182.28 ± 39.48	*NS*
**HDL-C** (mg/dL)^b^	41.1 (15.3 - 85.6)	37.2 (15.3 - 73.1)	44.5 (18.4 - 85.6)	*< 0.001*
**LDL-C** (mg/dL)^a^	107.06 ± 25.57	109.30 ± 24.56	104.97 ± 26.32	*0.005*
**HOMA-IR**^b^	2.58 (0.109 - 35.69)	2.50 (0.32 - 35.69)	2.65 (0.109 - 27.65)	*NS*
**Uric acid**	5.01 ± 1.15	5.63 ± 1.05	4.44 ± 0.92	*< 0.001*

a Data presented as mean plus or minus standard deviation;

b data presented as median (minimum to maximum);

^c^data presented as number (percent). WC: waist circumference; WHR: waist to hip ratio; SBP: systolic blood pressure; DBP: diastolic blood pressure; TGs: triglycerides; HDL-C: high density lipoprotein cholesterol; LDL-C: low density lipoprotein cholesterol; IR: insulin resistance; NS: not significant

**Figure 1:**
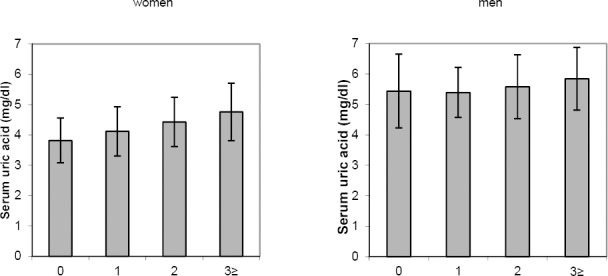
Serum uric acid levels according to the number of metabolic syndrome components and sex. Error bars are SDs

UA levels increased significantly with an increasing number of MS components in both genders (Figure 1). Post Hoc test confirmed that presence of 3 components or more of MS in males, and presence of even one of its components in females led to the above mentioned association.

UA levels of subjects with and without MS and every component of MS has been shown in Table 2. UA levels were significantly higher in subjects with MS, hypertension, central obesity, hypertriglyceridemia, and in females with hyperglycemia and low HDL.

**Table 2:** Comparison of uric acid levels between subjects with and without MS and its features

MS	+	-	P- value
All	5.25±1.12	4.84±1.11	<0.001
Men	5.84±1.03	5.47±1	<0.001
Women	4.76±0.95	4.22±0.83	<0.001

High BP	+	-	P- value
All	5.35±1.23	4.90±1.09	<0.001
Men	5.82±1.22	5.54±0.96	0.006
Women	4.72±0.94	4.37±0.90	<0.001

High WC	+	-	P- value
All	5.04±1.16	4.98±1.13	NS
Men	5.90±1.10	5.44±0.97	<0.001
Women	4.60±0.91	4.03±0.79	<0.001

High FBS	+	-	P- value
All	5.25±1.19	4.91±1.11	<0.001
Men	5.69±1.14	5.59±1.00	NS
Women	4.70±1.02	4.35±0.86	<0.001

High TG	+	-	P- value
All	5.31±1.13	4.80±1.11	<0.001
Men	5.79±1.06	5.48±1.03	<0.001
Women	4.72±0.93	4.29±0.87	<0.001

Low HDL	+	-	P- value
All	5.03±1.08	4.97±1.22	NS
Men	5.63±0.95	5.59±1.12	NS
Women	4.55±0.93	4.18±0.81	<0.001

NS: not significant

Subjects with hyperuricemia had higher prevalence of every component of MS with the exception of low HDL-C and a 3.23-fold higher risk of MS in comparison with normouricemic subjects. Among the MS components, hypertriglyceridemia demonstrated the strongest association with hyperuricemia (Table 3).

**Table 3:** Association of hyperuricemia with MS and its features

	UA (mg/L)		OR (95% CI)	P-value

	M<7, F<6 n=(%)	M≥7, F≥6 n= (%)		
High WC	568(56.3)	48(72.7)	2.07 (1.18-3.60)	0.01
High BP	250(25)	28(41.8)	2.16 (1.30-3.58)	0.004
High TG	405(40.1)	44(65.7)	2.85 (1.69-4.80)	<0.001
High FBS	293(29)	31(46.3)	2.11 (1.28-3.47)	0.004
Low HDL	657(65)	47(71.2)	1.33 (0.77-2.30)	NS
MS	376(37.6)	43(66.2)	3.23 (1.90-5.49)	<0.001

NS: not significant

In both genders, UA levels were positively and significantly correlated with WC, BP and TGs. UA levels were significantly and negatively correlated with HDL-C in females. UA continued to have a significant correlation with WC and TGs level in both genders and with HDL-C in females after adjusting for age, Cr and cholesterol. Otherwise, there was significant and negative correlation between UA levels and FBS in males after this adjustment.

Table 4 shows partial correlations between UA and different variables in 3 models. UA continued to have a significant correlation after adjusting for age and sex, except for diastolic blood pressure (DBP) and FBS. After further adjustment for Cr and cholesterol, there were no significant correlation between UA levels and HOMA-IR. After more adjustment for WC and BMI, UA had positive correlation just with TGs, HDL-C, and 2 hours blood glucose.

**Table 4:** Partial Correlation coefficients between uric acid and other variables

Variable	Unadjusted	Model 1	Model 2	Model 3
**SBP** (mmHg)	0.204^***^	0.118^***^	0.085^**^	0.025
**DBP** (mmHg)	0.158^***^	0.057	0.035	-0.029
**BMI** (kg/m^2^)	0.166^***^	0.307^***^	0.284^***^	-
**WC**(Cm)	0.341^***^	0.284^***^	0.252^***^	-
**WHR**	0.377^***^	0.171^***^	0.154^***^	0.056
**FBS** (mg/dL)	0.016	0.005	-0.033	-0.061
**2 hrs Blood glucose** (mg/dL)	0.078^**^	0.119^***^	0.099^**^	0.065^*^
**Chol** (mg/dL)	0.232^***^	0.234^***^	-	-
**TGs** (mg/dL)	0.357^***^	0.259^***^	0.181^***^	0.143^***^
**HDL-C** (mg/dL)	-0.263^***^	-0.109^**^	-0.144^***^	-0.092^**^
**Fasting Insulin**(μIU/mL)	0.083^**^	0.107^**^	0.077^*^	0.017
**HOMA-IR**	0.077^**^	0.086^**^	0.046	-0.016

Model1: Adjusted for age & gender Model2: Adjusted for age, gender, creatinin & cholesterol Model3: Adjusted for age, gender, creatinin, cholesterol, BMI & waist circumference

* P-value < 0.05;

** P-value < 0.01;

*** P-value < 0.001

The prevalence of MS according to UA quartiles has been shown in Table 5. The quartiles included serum UA levels less than 4.94, 4.94-5.58, 5.58-5.14, and more than 6.14 in males. It considered less than 3.77, 3.77-4.39, 4.39-5.10, and more than 5.10 in females. Prevalence of MS increased across UA quartiles in females; however the increasing trend began from second quartile in males.

**Table 5:** Prevalence of metabolic syndrome and odds ratio for metabolic syndrome according to the serum uric acid quartile

	Q1	Q2	Q3	Q4
**Whole**				
**Subjects (n)**	274	263	271	256
**Prevalence n (%)**	74(27)	83(31.6)	115(42.4)	147(57.4)
**Unadjusted**	1	0.802(0.553-1.165)	0.502(0.350-0.719)	0.274(0.191-0.395)
**Model 1**	1	1.021(0.877-1.189)	0.959(0.831-1.107)	0.890(0.771-1.027)
**Model 2**	1	0.916(0.771-1.088)	0.998(0.850-1.172)	1.052(0.900-1.230)
**Model 3**	1	1(0.999-1.001)	1(0.999-1.001)	1(1-1.001)
**Model 4**	1	1(0.999-1.001)	1(0.999-1.001)	1(0.999-1.001)
**Model 5**	1	1(1-1.001)	1(1-1.001)	1(1-1.001)
**Females**				
**Subjects (n)**	143	136	144	130
**Prevalence n (%)**	35(24.5)	48(35.3)	67(46.5)	78(60)
**Unadjusted**	1	0.594(0.354-0.998)	0.372(0.225-0.616)	0.216(0.129-0.363)
**Model 1**	1	1.123(0.759-1.664)	0.944(0.654-1.363)	0.826(0.570-1.197)
**Model 2**	1	0.719(0.453-1.141)	0.946(0.617-1.451)	0.984(0.643-1.506)
**Model 3**	1	0.999(0.997-1.001)	1(0.998-1.002)	1(0.998-1.002)
**Model 4**	1	1.002(0.999-1.004)	1(0.998-1.003)	1(0.998-1.003)
**Model 5**	1	1.001(1-1.002)	1.001(1-1.002)	1.001(1-1.003)
**Males**				
**Subjects (n)**	131	127	127	126
**Prevalence n (%)**	39(29.8)	35(27.6)	48(37.8)	69(54.8)
**Unadjusted**	1	1.114(0.649-1.912)	0.698(0.415-1.172)	0.350(0.210-0.585)
**Model 1**	1	0.706(0.473-1.054)	0.653(0.453-0.943)	0558(0.394-0.790)
**Model 2**	1	1.316(0.886-1.965)	1.349(0.943-1.931)	1.625(1.164-2.270)
**Model 3**	1	1.001(0.999-1.003)	1.001(1-1.003)	1.002(1.001-1.004)
**Model 4**	1	0.999(0.997-1.001)	0.999(0.997-1)	0.998(0.997-1)
**Model 5**	1	1(0.999-1.001)	1.001(0.999-1.002)	1(0.999-1.001)

Model1: Adjusted for age & gender Model2: Adjusted for age & gender & Creatinin Model3: Adjusted for age & gender & Cholesterol Model4: Adjusted for age & gender & Creatinin & Cholesterol Model5: Adjusted for age & gender & Waist Circumference

Using the lowest quartile of UA level as a reference, crude and adjusted multivariate odd’s ratios (ORs) were calculated according to various models. Association between UA level quartiles and MS using logistic regression analysis has been shown in Table 5. There were increases in ORs after adjustment for age and gender. After any adjustment for each individual variable in models, the association became insignificant. Cr, Cholesterol and WC were the most important factors in the association; as after adjustment for each of these factors, there were no significant multivariate ORs in both genders. There were no significant association between UA quartile groups and MS.

## 4. Discussion

Elevated serum UA level is associated with cardiovascular risk factors including hypertriglyceridemia, hypertension, obesity, and hyperglycemia. Presence of these factors together in the same patient is known as MS (Lin, Tsai, & Hsu, 2006). UA levels depend on UA renal excretion and cellular metabolism in the body. Age, gender, genetic factors and dietary habits may influence UA levels (Chiou et al., 2012).

The prevalence of hyperuricemia in the present study was far lower than in many related studies include studies on Iranian population (Sadr et al., 2009; Sari et al.2009; Poletto et al., 2011; Liu et al., 2011; Uaratanawong et al., 2011; Chuang et al., 2011). However, it was similar to a study conducted in Saudi Arabia (Al-Arfaj, 2001). Higher prevalence of hyperuricemia and higher UA levels in males than in females were confirmed in the current study; as they are reported in other comparable studies (Sui et al., 2008; Sadr et al., 2009; Sari et al..2009; Poletto et al., 2011; Liu et al., 2011; Uaratanawong et al., 2011; Chuang et al., 2011; Al-Arfaj, 2001; Lohsoonthorn, Dhanamun, &Williams et al., 2006; Cai et al., 2009). This finding was not unexpected since it is attributable to the effect of estrogen on UA excretion (Lin, Tsai, & Hsu, 2006). Estrogen therapy in males has been shown to decrease serum UA levels (Nicholls, Snaith, & Scott, 1973).

The Present study showed that UA levels were significantly higher in subjects who had increased number of MS components. This relationship was also significant in both genders. These findings have been reported in other studies (Conen et al., 2004; Solymoss et al., 2004; Desai et al., 2005).

UA levels were significantly higher in subjects with the MS compared to healthy individuals. The same relationship is affirmed in other studies (Reimann et al., 2008; Sui et al., 2008; Numata, et al., 2008). Hyperglycemic Females had higher UA levels. It has been shown that UA levels are higher in nondiabetic range of fasting serum glucose level and decreased after the onset of diabetes, particularly in diabetic males (Nan et al., 2008; Lin et al., 2004). Moreover, central obesity, hypertension and hypertriglyceridemia had significant association with hyperuricemia in both genders. Higher UA levels in subjects with central obesity could be mainly explained by impaired renal clearance of uric acid. In addition visceral fat obesity has been strongly associated with excess production of UA (Matsuura et al., 1998).

Although the association of UA and MS is not completely understood, it has been suggested that the relationship between UA levels and MS may be secondary to obesity, insulin resistance, and dyslipidemia (Meshkani, Zargari, & Larijani, 2010). On the other hand it has been proposed that UA may be a causative factor for MS. Hyperinsulinemia increases tubular sodium resorption that results in impaired UA excretion. So, normoglycemic subjects may have higher UA level in the presence of insulin resistance (Facchini et al., 1991). Hyperinsulinemia in subjects with MS decreases renal excretion of UA that contribute to hyperuricemia (Heinig et al., 2006). Insulin resistance is associated with high TGs level, high WC, and low HDL-C (Lin, Tsai, & Hsu, 2006). So, it can be suggested that higher UA levels in subjects with abnormal TGs, HDL-C level and WC may be caused by insulin resistance.

UA levels were positively correlated with WC and TGs level in both genders and were negatively correlated with HDL-C in females in the present study. Unexpectedly negative correlation between UA levels and FBS was found in males that had been also reported by Lu et al formerly (Lu et al., 2012). The Pearson’s correlation coefficient between UA levels and WC was the strongest following TGs level in both genders that suggests the abdominal obesity as a main determinant of hyperuricemia.

We found positive correlation between UA levels and MS and its components including TGs level, BP and WC in studied subjects. Independent correlation of TGs with UA levels has been reported by previous studies; but its underlying biological mechanism has not been elucidated. This association may be due to both environmental and genetic factors (Lin, Tsai, & Hsu, 2006; Fox et al., 1985).

After adjustment for different confounders, there was no significant correlation between UA levels and BP. As WC was a main determinant of UA levels in present study, it may be due to the effect of obesity on SBP and DBP. Negative correlation between UA levels and HDL-C is compatible with studies in Hangzhou and Taiwan (Cai et al., 2009; Chien et al., 2005). The lack of a significant correlation between UA levels, BP and FBS is in agreement with findings of previous studies (Lin, Tsai, & Hsu, 2006; Onat et al., 2006; Yoo et al., 2005; Lim et al., 2010). There are some reports about negative correlation of serum UA with blood glucose levels in diabetic patients; we found similar association for only male subjects in the present study (Tinahones et al., 2007; Choi & Ford, 2007).

An increase in the prevalence of MS with increasing UA levels was found in the present study that is consistent with previous studies performed on various ethnic groups. These studies have not explained that whether the relationship between UA and MS is independent or not (Lin, Tsai, & Hsu, 2006; Onat et al., 2006; Yoo et al; 2005). Positive association between MS severity and UA quartiles has been demonstrated in the present study that is in accordance with Hangzhou study (Cai et al., 2009). Association analysis has been limited to correlation between UA and other variables and comparing the MS prevalence in UA quartiles in most related studies.

The multivariate unadjusted ORs between UA quartiles and MS were less than 1 in the present study which was increased after adjustment for different variables in the models. This is in contrast with findings of Lim et al and Meshkani et al studies in which ORs more than 1 decreased after adjustment (Meshkani, Zargari, & Larijani, 2010; Lim et al., 2010). Low prevalence of hyperuricemia and high prevalence of MS among studied subjects may explain the difference.

When we added Cr, cholesterol levels and WC in adjustment model, the ORs of all quartiles was increased; but the relationship was not statistically significant with regards to confidence interval and P-values. Same results have been reported by the above two mentioned studies. Such results imply that the association between UA levels and MS is not independent, but is affected by various confounders such as physical activity and smoking. In Onat et al study, the ORs of the upper UA tertile were significantly decreased in both genders; but lost significance in males after adjustment for WC (Onat et al., 2006). Three prospective cohort studies in Korea, United States and Portugal have been suggested UA as an independent predictor of MS. They have shown that basal UA levels are associated with higher risk of MS in males and females (Gonçalves et al., 2012; Sui et al., 2008; Ryu et al., 2007). In another study performed in Taiwan the association has been found only in females (Yang et al., 2012).

The present study had some limitations include the cross sectional basis of its design and the number of studied subjects. Some confounders include physical activity; the use of diuretics and a diet habitually high in purines content were not considered. Other unknown confounders may also affect the results.

## 5. Conclusion

UA level is not a part of JIS criteria or other similar criteria for definition of MS. The association between UA levels and MS is not independent, but is affected by various confounders such as Cr and cholesterol levels, WC, physical activity and smoking. Results of the present study showed that UA levels are not an appropriate predictor of MS in Iranian population. Relatively low prevalence of hyperuricemia, low purine content of Iranian diet and high prevalence of MS may explain these results. More longitudinal studies are necessary to confirm the role of UA in MS occurrence.
